# An Electrochemical Impedance Spectroscopy System for Monitoring Pineapple Waste Saccharification

**DOI:** 10.3390/s16020188

**Published:** 2016-02-04

**Authors:** Claudia Conesa, Javier Ibáñez Civera, Lucía Seguí, Pedro Fito, Nicolás Laguarda-Miró

**Affiliations:** 1 Instituto de Ingeniería de Alimentos para el Desarrollo (IIAD), Universitat Politècnica de València, Camí de Vera s/n, 46022 Valencia, Spain; clcodo@upvnet.upv.es (C.C.); lusegil@upvnet.upv.es (L.S.); pfito@tal.upv.es (P.F.); 2 Centro de Reconocimiento Molecular y Desarrollo Tecnológico (IDM), Unidad Mixta Universitat Politècnica de València—Universitat de València, Camí de Vera s/n, 46022 Valencia, Spain; jibanyez@eln.upv.es

**Keywords:** electrochemical impedance spectroscopy, saccharification, monitoring, pineapple waste

## Abstract

Electrochemical impedance spectroscopy (EIS) has been used for monitoring the enzymatic pineapple waste hydrolysis process. The system employed consists of a device called Advanced Voltammetry, Impedance Spectroscopy & Potentiometry Analyzer (AVISPA) equipped with a specific software application and a stainless steel double needle electrode. EIS measurements were conducted at different saccharification time intervals: 0, 0.75, 1.5, 6, 12 and 24 h. Partial least squares (PLS) were used to model the relationship between the EIS measurements and the sugar determination by HPAEC-PAD. On the other hand, artificial neural networks: (multilayer feed forward architecture with quick propagation training algorithm and logistic-type transfer functions) gave the best results as predictive models for glucose, fructose, sucrose and total sugars. Coefficients of determination (R^2^) and root mean square errors of prediction (RMSEP) were determined as R^2^ > 0.944 and RMSEP < 1.782 for PLS and R^2^ > 0.973 and RMSEP < 0.486 for artificial neural networks (ANNs), respectively. Therefore, a combination of both an EIS-based technique and ANN models is suggested as a promising alternative to the traditional laboratory techniques for monitoring the pineapple waste saccharification step.

## 1. Introduction

Energy consumption has dramatically increased as a consequence of the world population growth and industrialization [1]. The depletion of fossil reserves and global warming and its consequences have become a matter of great concern [2]. In this global scenario, extensive research has been carried out on biofuels as a sustainable and environmental friendly alternative energy source to fossil fuels [3,4,5]. In fact, ethanol production from lignocellulosic biomass is reaching a huge interest and nowadays bioethanol is the most widely used liquid biofuel for motor vehicles [6] as it can be used either as pure fuel or be blended into gasoline (5%–10%) and even used as a gasoline additive, replacing methyl tertiary butyl ether (MTBE) which is potentially toxic to human health [7].

In the field of ethanol production from lignocellulosic biomass, industrial pineapple waste is particularly interesting as pineapple is the third most abundantly traded fruit worldwide and its waste represents about 50% (W/W) of the total processed fruit [8]. In addition, its waste contains high amounts fermentable sugars and potentially hydrolysable cellulose and hemicellulose [9,10,11].

Producing bioethanol from lignocellulosic biomass requires the hydrolysis of a part of the cellulose (a polymer of D-glucose units linked by β-1,4-glycosidic bonds) and hemicellulose (different polymers of pentoses, hexoses and uronic acids) into fermentable sugars by fungal enzymatic complexes: cellulases and hemicellulases, respectively [12]. However, saccharification is the most complex step in the bioethanol production process [13]. In fact, the mechanism of the enzymatic hydrolysis and the relationships between the enzyme and the substrate structure are particularly complex [14]. Moreover, several authors have suggested that some sugars released during saccharification could inhibit the hydrolysis reaction, specifically cellobiose and to a lesser extent glucose [15,16,17,18]. Therefore, monitoring enzymatic saccharification is of capital importance for maximizing sugar yields and ethanol production.

Nowadays, different chromatographic techniques such as high pressure liquid chromatography (HPLC) using various columns and detectors [19,20] or gas chromatography-mass spectrometry (GC-MS) [21,22] have been successfully used for carbohydrate analyses. Specifically, high-performance anion-exchange chromatography with pulsed amperometric detection (HPAEC-PAD) is considered as the most highly sensitive and reliable method for all sugars [23]. However, these conventional techniques are destructive, time consuming, require specialized sample preparation with hazardous chemicals and are labor intensive [24]. In contrast, electrochemical impedance spectroscopy (EIS) is a non-destructive, rapid, simple, real-time analysis methid, with no use of toxic reagents and with relatively reduced operational costs. EIS allows the analysis of the properties of materials and systems by applying alternate electric signals (voltage or current) of different frequencies to them and measuring the corresponding electric output signal (current or voltage) [25,26]. EIS has been shown to be a powerful technique for monitoring the effects of industrial processing methods such as heat treatments [27], freezing [28] or cold injuries [29] on agricultural products (fruits and vegetables).

For further data processing, EIS requires powerful mathematical and statistical tools in order to get robust and reliable responses from the huge amount of data generated. This is the specific case of principal component analysis (PCA) and PLS that have been shown as excellent mathematical tools for this kind of data [30,31], but also the case of ANNs that are widely used to perform sample classification [32,33]. In fact, ANNs outperform these methods due to their enormous flexibility and adaptive capacity, accurate fit to nonlinear systems and ability to learn from their mistakes. In addition, ANN-based classification/modeling systems are clear, easy to use, have a low computational burden and their algorithms are easily implementable on a PC or microprocessor. Potential applications of ANNs in microprocessors are of particular interest as they allow the design of portable devices that can *in situ* analyses, with great flexibility, a wide range of applications, easy operation and low power consumption. For such applications, [34] other authors [35,36] have developed simplified ANN versions by simplifying the architecture and learning equations and further reducing the computational costs. Thus, the system has less computational requirements, being faster to program and easier to operate, while offering almost the same reliability [37].

In previous studies, a system consisting of a stainless steel double needle electrode associated with specific electronic equipment was designed. This device was called Amperometry, Voltammetry, Impedance Spectroscopy and Potentiometry Analyzer (AVISPA) and it allowed the implementation of EIS analyses on previously prepared samples [38]. This previous step in this research showed that a combination of both EIS-based technique and the designed ANN could be considered a promising alternative to traditional laboratory techniques for identifying and quantifying glucose, fructose and sucrose added to pineapple wastes. Therefore, this study aims to verify that natural enzymatic saccharification of pineapple waste can be accurately monitored by using an EIS system.

## 2. Experimental

### 2.1. Sample Preparation

Pineapple (*Ananas comosus* L. cv. “MD-2”) fruit selection was based on the visual absence of external defects. Next, a NaClO (0.1%) solution was used to wash the pineapples. Then, the crown was removed and the pulp was separated from the rest of the fruit using a pineapple cutter. Next, peel and core (waste) were cut into smaller pieces and crushed in a blender (Avance Collection Blender HR2097/00 800 W, Philips, Amsterdam, The Netherlands). Finally, the mashed product was frozen and stored at −22 °C. Enzymatic hydrolysis was carried out by adding 0.4% (w/v) of cellulase (1.13 U/mg solid, Sigma-Aldrich Química SL, Madrid, Spain) and 0.4% (w/v) of hemicellulase (1.5 U/mg solid, Sigma-Aldrich Química SL, Madrid, Spain) from *Aspergillus niger* to 40 g of thawed pineapple waste in a 100 mL glass beaker. Samples were placed in a thermostatic bath (Precisdig, JP SELECTA S.A., Barcelona, Spain) at 40 °C for 24 h.

### 2.2. Electrochemical Impedance Spectroscopy Equipment

EIS measurements were carried out using a measurement system developed by the Group of Electronic Development and Printed Sensors (GED+PS) belonging to the Centro de Reconocimiento Molecular y Desarrollo Tecnológico (IDM) at the Universitat Politècnica de València (UPV). This system consists of a device called Advanced Voltammetry, Impedance Spectroscopy & Potentiometry Analyzer (AVISPA) associated to a specific software application that is able to apply different sinusoidal voltage signals with amplitudes up to 1 Vpp and frequency sweep from 0.01 Hz to 10 MHz using up to 32 current scales [38]. It means that the frequency range, the number of the sweep frequencies, the current scale and the amplitude of the sine wave applied to the sensor can be completely configured by means of the software.

The applied sensor was the same as the one previously used in the identification and quantification of sugars added to pineapple waste [38], consisting of a 1.5 cm in length and 1 mm in diameter double needle electrode (working and counter electrodes) made of stainless steel and encapsulated in epoxy resin to assure a constant separation of 1 cm between the needles.

### 2.3. Electrochemical Impedance Spectroscopy Measurements

**Figure 1:**
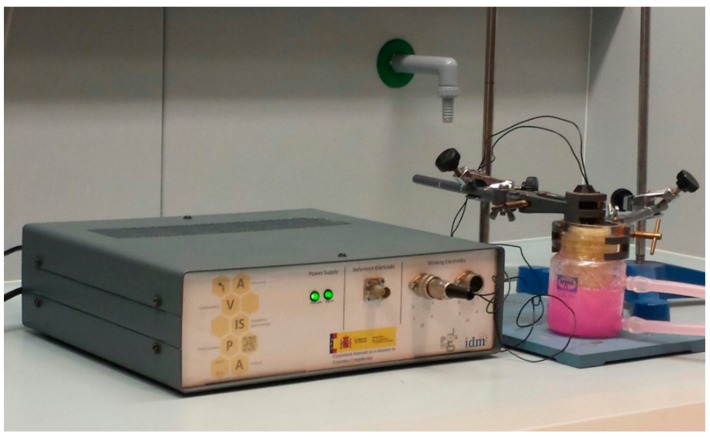
A view of the experimental set-up.

EIS measures were conducted in saccharified pineapple waste samples at different time intervals: 0, 0.75, 1.5, 6, 12 and 24 h. The sensor was completely introduced into the samples (1.5 cm) in order to assure a constant contact surface between the electrode and the samples. A thermostatic bath (PolyScience^®^, Niles, IL, USA) was used to conduct assays in triplicate at 25 °C for an total of 54 EIS measurements (Figure 1).

Memory effects were minimized by a random selection of the analyzed samples. The use of the same homogenous pineapple waste for all the analyses avoided any effect of potential interfering compounds in the samples. The AVISPA device started measuring the response plot of the applied EIS signal and sending it to a PC to calculate modulus and phase values and generate the corresponding plot. This step was repeated for all the frequencies in the sequence in order to have a complete graph of the sample response.

### 2.4. High Performance Anion-Exchange Chromatography-Pulsed Amperometric Detection (HPAEC-PAD) Analyses

Sugars present in the liquid phase of the same pineapple waste samples were measured at identical time intervals as stated before by using an IC chromatograph system (Metrohm, Herisau, Switzerland) equipped with a 716 Compact module and an ICnet 2.0 software program for interpreting the results. A three-step PAD setting was applied with the following path intervals (ms) and potentials (V): t_1_: 400/E_1_ = +0.05 (detection); t_2_: 200/E_2_ = +0.75 (cleaning); t_3_: 400/E_3_ = −0.15 (regeneration). The column used was a Metrosep Carb 1 250/4.6 column (250 mmL × 4.6 mmID) coupled to a guard column. Analyses were done at 32 °C, 8.8 MPa, injection volume: 20 μL and using sodium hydroxide 0.1 M as the mobile phase (1 mL/min). Chromatographic measurements required filtration of the liquid (0.45 μm nylon filter) and dilution of the resulting filtered sample (1:2000 v/v in bidistilled water). High-purity (≥99%) standards of glucose, fructose and sucrose (Sigma-Aldrich Química SL) were used to prepare standard calibration curves (2.5, 5, 10, 15, 25 and 50 ppm). All the determinations were carried out in triplicate.

### 2.5. Statistical Analysis and Modeling

Statgraphics Centurion XVI^®^ (Manugistics Inc.; Rockville, MD, USA) was used for analyzing the HPAEC-PAD results. Statistically significant differences between sugar yields during saccharification were determined using one-way analyses of variance (ANOVA) with Multiple Range Test (95% confidence level).

The electrochemical data were analyzed using multivariate techniques, applying the software SOLO^©^ (Eigenvector Research, Inc., Manson, WA, USA). Principal Component Analyses (PCA) were carried out with data obtained from the specific impedance modulus and phase data obtained in the frequency range in which the sensor showed the highest sensitivity. Partial Least Squares (PLS) were used to model the relationship between the array of dependent variables Y (EIS measurements) and the array of independent variables X (sugar determination by HPAEC-PAD). According to [30,39], PLS prediction models were created using the 66% of the experimental data (calibration set) and the model was then validated with the remaining 34% (validation set). The accuracy was given by the root mean square error of prediction (RMSEP) and the coefficient of determination (R^2^).

Alyuda Neurointelligence 2.2^©^ (Alyuda Research Inc., Los Altos, CA, USA) was used to design and implement the ANN. This software enables users to select the main network parameters (e.g., the training algorithm type, the number of neurons in hidden layer and transference functions). It also randomly divides the experimental data into the following three sets: training (70%), validation (15%) and test (15%) data [35,40,41]. The training data was used to compute the network parameters and the testing data, to ensure robustness of the network parameters. A proportional number of nodes in the network architecture [42], cross validation and early-stopping in the training phase were used in order to avoid the “overfitting” phenomenon, so that the difference between training and validation mean square errors was minimal. Therefore, multi-layer feedforward networks with a single hidden layer were used to predict glucose, fructose, sucrose and total sugars evolution during saccharification process. Quick propagation training (a modified version of the back propagation algorithm), was selected for fitting the network [43]. The optimal network topology (architecture and number neurons in each hidden layer) was selected by testing several artificial neural network structures. Similarly, several trials suggested the selection of logistic-type transfer functions for both output layer neurons and hidden nodes. As described before, the performance of the designed ANN models was assessed on the basis of RMSEP and R^2^ between the predicted values of the network and the experimental data.

## 3. Results and Discussion

### 3.1. Sugar Determination by HPAEC-PAD

**Figure 2:**
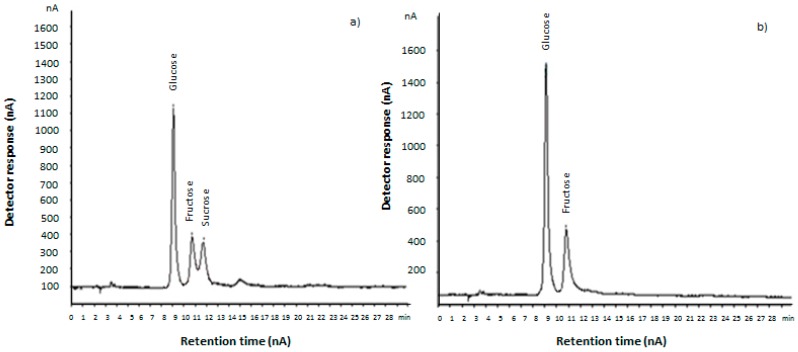
HPAEC-PAD chromatograms (dilution 1:2000 v/v) for (**a**) pineapple waste before saccharification (0 h); and (**b**) saccharified pineapple waste (24 h).

Table 1 summarizes the evolution of the sugar profile of pineapple waste throughout the enzymatic treatment. To that end, sugars present in the hydrolyzed samples (0 h–24 h) were identified and quantified through the chromatograms obtained from the HPAEC-PAD analysis of the liquid phase (Figure 2).

As shown in Table 1, glucose, fructose and sucrose were identified in the liquid phase of the pineapple waste before saccharification (0 h). Glucose and fructose were present in similar amounts, while sucrose concentration was slightly lower. These results are similar to those reported in [44,45].

**Table 1:** Sugar profile of pineapple waste during saccharification at different time intervals (0 h to 24 h). Values correspond to the average of three replicates (Standard deviation).

Saccharification Time (h)	Glucose (g/L)	Fructose (g/L)	Sucrose (g/L)	Total Sugars ^1^ (g/L)
**0**	33.5 (1.2) ^a^	33.18 (1.1) ^a^	28.1 (0.3) ^e^	94.8 (0.3) ^a^
**0.75**	36.4 (1.6) ^b^	36.69 (0.15) ^b^	20.6 (1.5) ^d^	95.7 (0.2) ^b^
**1.5**	36.1 (0.9) ^b^	41.3 (0.5) ^c^	19.0 (0.7) ^c^	96.5 (1.4) ^b^
**6**	44.3 (0.5) ^c^	51.3 (1.1) ^d^	9.4 (0.3) ^b^	105.0 (0.9) ^c^
**12**	47.63 (1.07) ^d^	58.9 (1.2) ^e^	0.0 (0.0) ^a^	107.0 (0.7) ^d^
**24**	48.8 (0.8) ^d^	60 (2) ^e^	0.0 (0.0) ^a^	108 (3) ^d^

^a,b,c,d,e^ Similar lowercase letters indicate statistically homogeneous groups with a confidence level of 95%; ^1^ Total Sugars obtained by adding glucose, fructose and sucrose contents.

The enzymatic treatment produced a statistically significant increase in the glucose (45%) and fructose (81%) contents, whereas the sucrose content decreased and eventually disappeared as the saccharification process proceeded. Specifically, glucose release is probably due to the action of the fungal cellulase complex that consists of three groups of enzymes: (1) the endoglucanases (EC 3.2.1.4.) act by randomly hydrolyzing the internal glycosidic linkages of the cellulose chain; (2) the cellobiohydrolases, also known as exoglucanases (EC 3.2.1.74), act on the ends of the chains, releasing glucose monomers, cellobiose and low molecular weight oligosaccharides; and (3) the β-glucosidases (EC 3.2.1.21) convert cellobiose to glucose [46,47]. On the contrary, the sucrose decrease and fructose increase would not be the result of the enzymatic action but rather of sucrose inversion [48], considering the acid pH of the medium and the fact that selected enzymes are not potentially capable of reversing sucrose. As a consequence, total sugars in the hydrolyzed samples (24 h) increased by 24% compared to the original one (0 h).

### 3.2. Electrochemical Impedance Spectroscopy Measurements

**Figure 3:**
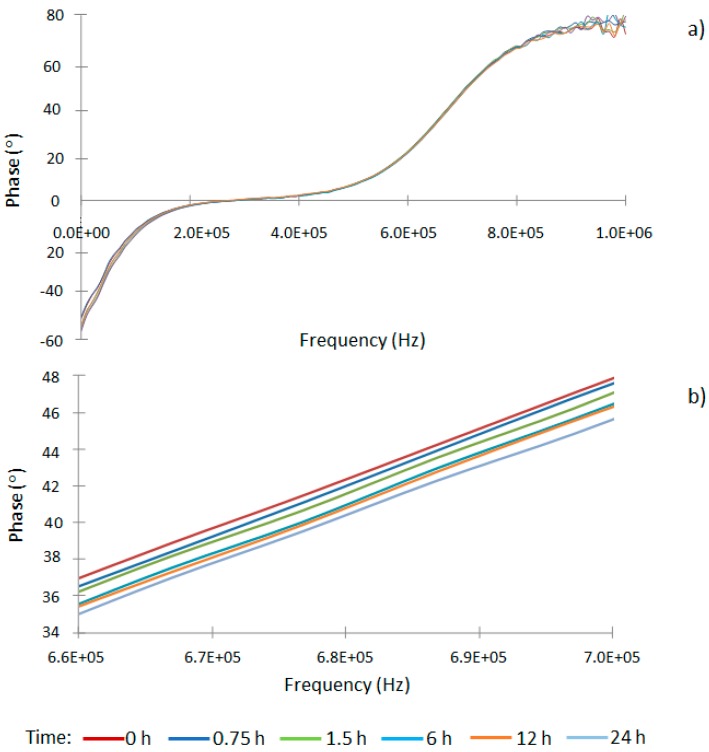
Phase data for pineapple waste saccharification at different time intervals (0 to 24 h) for (**a**) the complete analyzed frequency range; and (**b**) the studied frequency range (6.6 × 10^5^ Hz–7.0 × 10^5^ Hz).

For each analyzed sample, the AVISPA device generated 200 datasets corresponding to the modulus and phase of the 100 applied frequencies (between 1 and 10^6^ Hz) at 0, 0.75, 1.5, 6, 12 h and 24 h since the saccharification process started. The graph of the modulus showed no relevant information, whereas the representation of the phase revealed that frequencies between 6.5 × 10^5^ Hz and 7.0 × 10^5^ Hz (6 frequency data) were the ones that showed the highest sensitivity to total sugars along the saccharification process (Figure 3). Consequently, this frequency range was selected for further data treatment and mathematical modeling.

**Figure 4:**
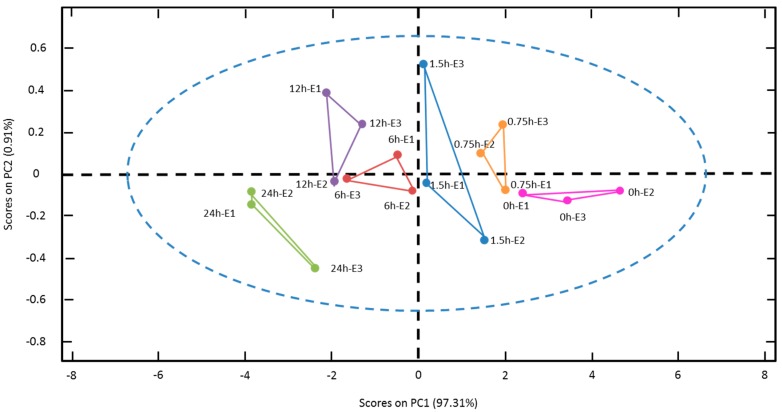
Principal component analyses (PCA) biplot for EIS phase data (6.6 × 10^5^ Hz–7.0 × 10^5^ Hz) during pineapple waste saccharification (0 to 24 h). R1-3: average of each replicate. The blue ellipsis indicates 95% confidence level.

PCA bi-dimensional graphic analyses showed a high percentage of the total variability (98.22%) being explained just with the first two components (Figure 4). The first component (PC1) and second component (PC2) explained 97.31% and 0.91% of the graphic variability, respectively. Therefore, the results indicate that variation of total sugars can be discriminated in the studied ranges with only one principal component. Obviously, this PC1 is directly correlated to the time variable in the saccharification process.

Since the PCA analysis demonstrated the ability of the designed EIS system to discriminate total sugar concentrations, PLS analyses were performed to predict glucose, fructose and sucrose concentrations from EIS measurements. Based to the results, models for glucose, fructose, sucrose and total sugars were built in the range of 0 to 24 h. Meanwhile the time range used for sucrose was just from 0 to 12 h due to its exhaustion.

The obtained correlations for the studied sugars are shown in Table 2. These results demonstrated accurate fitting between experimental and predicted data. Consequently, the designed models can be considered statistically valid. Moreover the PLS analyses showed that just one latent variable is enough to generate reliable mathematical models for the three analyzed sugars. Consequently, the prediction models could be simple and accurate as just one frequency data is enough to quantify these sugars along the saccharification process.

**Table 2:** Statistical values of Partial Least Square (PLS) discriminant analysis for the quantification of the studied fermentable sugars for EIS phase data from 6.6 × 10^5^ Hz–7.0 × 10^5^ Hz. (R^2^: coefficient of determination; RMSEP: Root Mean Square Error of Prediction; LV: Latent Variables).

Sugars	Statistics		
	R^2^	RMSEP	LV
Glucose	0.955	1.306	1
Fructose	0.970	1.782	1
Sucrose	0.975	1.645	1
Total Sugars	0.944	1.353	2

In the present work, artificial neural networks (ANNs) were studied as an alternative modeling method to PLS analyses. Thus, different multilayer feed forward net architectures with quick propagation training algorithms and logistic-type transfer functions were tested. Specifically, a (6-5-1) architecture was designed for fructose that means six input nodes (corresponding to the six analyzed frequencies) connected to a 5-node hidden layer and a final output layer. For glucose, sucrose and total sugars, (6-4-1), (6-9-1) and (6-1-1) architectures were selected, respectively.

**Figure 5:**
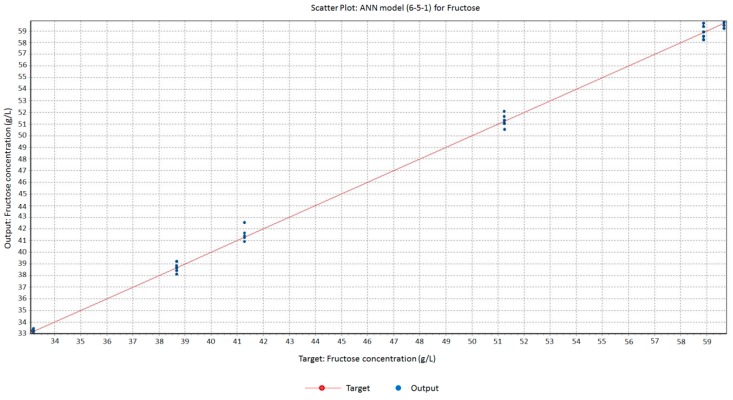
Scatter plot showing the relationship between analyzed (HPAEC-PAD) and predicted (ANN model) fructose concentrations in saccharified pineapple waste (g/L) for the studied EIS phase data (6.6 × 10^5^ Hz–7.0 × 10^5^ Hz).

Table 3 shows the mathematical models obtained for the corresponding ANN training, validation and test phase and Figure 5 shows the regression line obtained for fructose. As indicated, at least R^2^ ≥ 0.991 and RMSEP ≤ 0.901 were obtained for all the test phases. Thus, accurate and reliable models for determining sugar concentrations depending on EIS measurements were designed. Considering R^2^ and RMSEP parameters for both PLS and ANN models, a better fit was obtained by ANN than by PLS for all the studied sugars.

**Table 3:** Artificial neural network (ANN) results for the studied fermentable sugars for EIS phase data from 6.6 × 10^5^ Hz–7.0 × 10^5^ Hz (R^2^: coefficient of determination; RMSE: Root Mean Square Error).

	R^2^	RMSEP
**Glucose**		
Training	0.970	0.686
Validation	0.995	0.251
Test	0.998	0.233
**Fructose**		
Training	0.986	0.414
Validation	0.998	0.309
Test	0.996	0.486
**Sucrose**		
Training	0.995	0.666
Validation	0.987	1.206
Test	0.998	0.901
**Total Sugars**		
Training	0.989	0.420
Validation	0.991	0.298
Test	0.991	0.333

## 4. Conclusions

Pineapple industrial waste is generating increasing worldwide interest as an alternative energy source because of its large volume and useful biochemical properties that allow for high efficiency in bioethanol production. However, this raw material must be previously hydrolyzed and an accurate monitoring of this process is of interest as enzymatic hydrolysis is a particularly complex process. In this study, the validation of an EIS-based method to monitor the saccharification process of industrial pineapple waste is introduced as an innovative analytical procedure. The use of the AVISPA device associated to a stainless steel double needle sensor allowed the development of electrochemical measurements and further comparison between EIS and HPAEC-PAD results. Statistical tools such as PLS and ANN allowed the design of robust and reliable mathematical prediction models for glucose, fructose, glucose and total sugars (R^2^ > 0.970 and RMSEP < 1.206). Therefore, the introduced EIS-based technique combined with ANN models is validated and it is suggested as an easy, non-destructive and economic alternative to the traditional laboratory techniques for sugar monitoring in saccharification processes.
